# Prognostic value of a novel glycolysis-related gene expression signature for gastrointestinal cancer in the Asian population

**DOI:** 10.1186/s12935-021-01857-4

**Published:** 2021-03-04

**Authors:** Rong Xia, Hua Tang, Jiemiao Shen, Shuyu Xu, Yinyin Liang, Yuxin Zhang, Xing Gong, Yue Min, Di Zhang, Chenzhe Tao, Shoulin Wang, Yi Zhang, Jinyou Yang, Chao Wang

**Affiliations:** 1 Key Lab of Modern Toxicology of Ministry of Education, Center for Global Health, School of Public Health, Nanjing Medical University, 101 Longmian Avenue, Nanjing, 211166 People’s Republic of China; 2 State Key Lab of Reproductive Medicine, Institute of Toxicology, Nanjing Medical University, 101 Longmian Avenue, Nanjing, 211166 People’s Republic of China; 3 Department of General Surgery, Tongling People’s Hospital, 468 Bijiashan Road, Tongling, Anhui Province 244000 People’s Republic of China; 4 The First Clinical Medical College of Nanjing Medical University, Nanjing, 211166 People’s Republic of China; 5 Department of Colorectal Surgery, the First Affiliated Hospital of Nanjing Medical University, Nanjing, 210000 People’s Republic of China; 6 Department of Clinical Medicine and Rehabilitation, Jiangsu College of Nursing, 9 Keji Road, Huai’an, 223005 People’s Republic of China

**Keywords:** Gastrointestinal cancer, Glycolysis-related genes, Prognosis, Asian patients

## Abstract

**Background:**

Globally, gastrointestinal (GI) cancer is one of the most prevalent malignant tumors. However, studies have not established glycolysis-related gene signatures that can be used to construct accurate prognostic models for GI cancers in the Asian population. Herein, we aimed at establishing a novel glycolysis-related gene expression signature to predict the prognosis of GI cancers.

**Methods:**

First, we evaluated the mRNA expression profiles and the corresponding clinical data of 296 Asian GI cancer patients in The Cancer Genome Atlas (TCGA) database (TCGA-LIHC, TCGA-STAD, TCGA-ESCA, TCGA-PAAD, TCGA-COAD, TCGA-CHOL and TCGA-READ). Differentially expressed mRNAs between GI tumors and normal tissues were investigated. Gene Set Enrichment Analysis (GSEA) was performed to identify glycolysis-related genes. Then, univariate, LASSO regression and multivariate Cox regression analyses were performed to establish a key prognostic glycolysis-related gene expression signature. The Kaplan-Meier and receiver operating characteristic (ROC) curves were used to evaluate the efficiency and accuracy of survival prediction. Finally, a risk score to predict the prognosis of GI cancers was calculated and validated using the TCGA data sets. Furthermore, this risk score was verified in two Gene Expression Omnibus (GEO) data sets (GSE116174 and GSE84433) and in 28 pairs of tissue samples.

**Results:**

Prognosis-related genes (NUP85, HAX1, GNPDA1, HDLBP and GPD1) among the differentially expressed glycolysis-related genes were screened and identified. The five-gene expression signature was used to assign patients into high- and low-risk groups (*p* < 0.05) and it showed a satisfactory prognostic value for overall survival (OS, *p* = 6.383 × 10^–6^). The ROC curve analysis revealed that this model has a high sensitivity and specificity (0.757 at 5 years). Besides, stratification analysis showed that the prognostic value of the five-gene signature was independent of other clinical characteristics, and it could markedly discriminate between GI tumor tissues and normal tissues. Finally, the expression levels of the five prognosis-related genes in the clinical tissue samples were consistent with the results from the TCGA data sets.

**Conclusions:**

Based on the five glycolysis-related genes (NUP85, HAX1, GNPDA1, HDLBP and GPD1), and in combination with clinical characteristics, this model can independently predict the OS of GI cancers in Asian patients.

**Supplementary Information:**

The online version contains supplementary material available at 10.1186/s12935-021-01857-4.

## Background

Globally, cancers of the gastrointestinal (GI) tract, including those that originate from cells in the esophagus, stomach, exocrine pancreas, liver, gallbladder, biliary tract, small intestines, colon, rectum and anus, are associated with a high prevalence and mortality rate [1]. The prevalence of GI cancers, such as liver and gastric cancers, in Asia is higher than in North America or Europe [2]. The pathogenic factors for GI cancer are complex and include chronic inflammation, infection, environmental carcinogens and genetic susceptibility [3]. Currently, the main therapeutic options for GI cancers are surgical resection, radiotherapy and chemotherapy among others. However, the postoperative survival rate is still low [4]. Currently, the prognosis of GI cancer patients relies on traditionally recognized prognostic factors, such as pathological staging, histological grade and immunohistochemical studies of molecular markers [5]. Prognostic indicators are of great significance in developing new treatment strategies, therefore, independent indicators for better prognosis should be urgently established.

The tumor microenvironment is highly involved in the occurrence and development of malignant tumors, and is closely associated with energy metabolism. Mitochondrial oxidative phosphorylation and glycolysis are the two major pathways for cellular energy production [6]. Compared to normal cells, even under normoxia conditions, cancer cells mainly rely on glycolysis to produce the energy required for cellular processes [7]. This phenomenon is regarded as a landmark event in the process of tumor formation. Glycolysis and its related genes play a very essential role in the development of GI cancers [8]. Glucose metabolism, including glycolysis and hexosamine synthesis, is abnormally activated in liver cancer, leading to enhanced malignant phenotypes [9]. Moreover, in gastric cancer, the energy needs of tumor cells are achieved through glycolysis [10]. Enhanced glycolysis has also been shown to promote the proliferation and metastasis of colorectal cancer cells [11]. Currently, the mechanisms through which key enzymes and glycolysis-related genes in tumor metabolic pathways are regulated have not been elucidated. Therefore, elucidating the mechanisms through which metabolic remodeling occurs in tumors is of great clinical significance for the accurate diagnosis and treatment of GI cancers. So far, prognostic prediction models based on glycolytic genes have only been reported in liver and colon cancers [12, 13]. For the first time, we established a risk prediction model that is based on glycolytic genes for seven common tumors of the digestive tract to assist in identifying risky patients and follow-up to improve treatment outcomes in the Asian population.

In recent years, various risk prediction models that are based on gene expression data, such as autophagy-related genes prognosis prediction models [14], immune-related genes prognosis prediction models [15] and inflammation-related genes prognosis prediction models [16], are widely applied in the clinical prediction of patient survival. Elevating aerobic glycolysis and dependence on glycolysis to produce energy is one of the main metabolic characteristics of cancer [17]. Attempts have been made to target tumors by inhibiting the activity of key enzymes in the tumor glycolytic pathway. It has been reported that inhibiting the glycolytic pathway in tumors can effectively suppress the proliferation of tumor cells, and even play a role in killing tumor cells [18]. However, inhibition of a single target may be insufficient in suppressing tumor proliferation and may even cause drug resistance. For example, NRF2 has dual roles in cancer [19, 20]. The antioxidant function of NRF2 is important in protecting against cancer initiation and progression. Based on such a protective effect, numerous chemopreventive compounds that can activate NRF2 have been identified [21–23]. Besides, NRF2 can also exert cancer-promoting effects [24]. Several NRF2 target genes are involved in drug resistance [22]. Elevated NRF2 levels have been correlated with chemoresistance in cancer cells [20, 25–28]. In addition, the KRAS oncogene, a critical driver of multiple cancers, is also an important target for cancer therapy. Studies have reported that oncogenic KRAS alters glucose and glutamine metabolism to support pancreatic ductal adenocarcinoma cell proliferation [29–32]. KRAS upregulates stress-granule formation, which is involved in chemoresistance [32–34]. Because of the multiple functions of a single gene, it is insufficient to target gene for cancer therapy. Therefore, the therapeutic potential of combined treatment and predictors of multiple glycolytic enzyme targets should be studied [35]. In this study, we aimed at elucidating the relationship between glycolysis-related genes and clinical-related indicators from the entire Asian GI tumor, and to establish a more accurate prognostic model that is based on glycolysis-related genes. From the Cancer Genome Atlas (TCGA) database, we identified a glycolytic associated five-gene signature that is closely related to overall survival (OS) of GI cancer patients in the Asian population. Based these five genes, a prognostic prediction model was constructed and was shown to accurately predict and monitor the prognosis for GI cancers in the Asian population.

## Methods

### Data collection and mRNA expression dataset

The mRNA expression profiles and the corresponding clinical data for 296 Asian GI cancer patients were obtained from the TCGA database (http://cancergenome.nih.gov/). These patients were; 158 LIHC patients, 74 STAD patients, 38 ESCA patients, 11 PAAD patients, 11 COAD patients, 3 CHOL patients and 1 READ patients (TCGA-LIHC, TCGA-STAD, TCGA-ESCA, TCGA-PAAD, TCGA-COAD, TCGA-CHOL and TCGA-READ). Their detailed clinical information is summarized in Table 1. Various glycolysis-related genes were obtained from Molecular Signatures Database v7.0 (MSigDB) (https://software.broadinstitute.org/gsea/msigdb/index.jsp).

**Table 1:** Clinical pathological parameters of Asian patients with gastrointestinal cancer in this research

Tumor type	Clinical characteristic		N (%)
Liver hepatocellular carcinoma (LIHC)	Age (years)	> 65	36 (22.78)
		≤ 65	122 (77.22)
	Gender	Male	124 (78.48)
		Female	34 (21.52)
	Stage	I–II stage	151 (95.57)
		III–IV stage	7 (4.43)
	Vital status	Alive	114 (72.15)
		Dead	44 (27.85)
Stomach adenocarcinoma (STAD)	Age (years)	> 65	41 (55.41)
		≤ 65	33 (44.59)
	Gender	Male	49 (66.22)
		Female	25 (33.78)
	Stage	I- II stage	43 (58.11)
		III–IV stage	29 (39.19)
		Not reported	2 (2.70)
	Vital status	Alive	54 (72.97)
		Dead	19 (25.68)
		Not reported	1 (1.35)
Esophageal carcinoma (ESCA)	Age (years)	> 65	6 (15.79)
		≤ 65	32 (84.21)
	Gender	Male	35 (92.11)
		Female	3 (7.89)
	Stage	I–II stage	11 (28.95)
		III–IV stage	6 (15.79)
		Not reported	21 (55.26)
	Vital status	Alive	31 (81.58)
		Dead	7 (18.42)
Pancreatic adenocarcinoma (PAAD)	Age (years)	> 65	4 (36.36)
		≤ 65	7 (63.64)
	Gender	Male	5 (45.45)
		Female	6 (54.55)
	Stage	I–II stage	11 (100.00)
		III–IV stage	0 (0.00)
	Vital status	Alive	6 (54.55)
		Dead	5 (45.45)
Colon adenocarcinoma (COAD)	Age (years)	> 65	2 (18.18)
		≤ 65	9 (81.82)
	Gender	Male	8 (72.73)
		Female	3 (27.27)
	Stage	I–II stage	10 (90.91)
		III–IV stage	1 (9.09)
	Vital status	Alive	9 (81.82)
		Dead	2 (18.18)
Cholangiocarcinoma (CHOL)	Age (years)	> 65	2 (66.67)
		≤ 65	1 (33.33)
	Gender	Male	2 (66.67)
		Female	1 (33.33)
	Stage	I–II stage	3 (100.00)
		III–IV stage	0 (0.00)
	Vital status	Alive	1 (33.33)
		Dead	2 (66.67)
Rectum adenocarcinoma (READ)	Age (years)	> 65	0 (0.00)
		≤ 65	1 (100.00)
	Gender	Male	1 (100.00)
		Female	0 (0.00)
	Stage	I–II stage	1 (100.00)
		III–IV stage	0 (0.00)
	Vital status	Alive	1 (100.00)
		Dead	0 (0.00)

### Gene set enrichment analysis (GSEA)

GSEA analysis was performed using the GSEA software v4.0.1 and “h.all.v7.1.symbols.gmt” (http://www.broadinstitute.org/gsea) to evaluate whether the defined gene sets showed statistically significant differences between the tumor and normal tissues. *p* ≤ 0.05 and false discovery rate (FDR) < 0.25 were the criteria for identifying significantly enriched gene sets in GSEA.

### Prognostic signature construction

Raw mRNA expression data were normalized by [log2 (data + 1)] for further statistical analysis. Univariate Cox regression was used to screen and analyze the genes affecting the OS of patients (*p* < 0.05). Then, LASSO Cox regression and multivariate Cox proportional hazards regression models were used to identify and analyze the prognostic genes in order to establish a predictive model**.** The selected mRNAs were classified into two types; hazard ratio (HR) > 1 was the risk type while hazard ratio (HR) < 1 was the protective type. Based on the mRNA expression and coefficients as derived from the multivariate Cox proportional hazards regression analysis, a prognostic risk score formula was established. The risk score formula was: Risk score = expression of gene_1_ × β_1_gene_1_ + expression of gene_2_ × β_2_gene_2_ + …expression of gene_n_ × β_n_gene_n_ (β represents the regression coefficient of each mRNA).

### Sample collection and validation of the expression of glycolysis-related genes at mRNA and protein levels

The Institutional Review Board of Nanjing Medical University and the Ethical committee of the Tongling People’s Hospital approved this study (ethical review No. 2019-008). All study participants were required to sign an informed consent before enrollment. Twenty-eight paired GI tumors and adjacent non-tumor tissues were collected from patients at the Tongling People's Hospital from 2018 to 2019. All the patients had not received chemotherapy or radiotherapy before surgery. The obtained tissues included 8 paired COAD tissues, 5 paired READ tissues and 15 paired STAD tissues. All tissue samples were rapidly frozen and stored in liquid nitrogen until RNA extraction. Total RNA was extracted and subjected to reverse transcription followed by Real-time quantitative polymerase chain reactions (qRT-PCR), as previously described [36]. The primer sequences were: The forward primer for GAPDH was CCTTCCGTGTCCCCACT while its reverse primer was GCCTGCTTCACCACCTTC; the forward primer for NUP85 was CATTGAGCGGATACCTCTG while its reverse primer was GACGGCTTTCATGGCTAA; The forward primer for GPD1 was TCTTTGGGGAGCAGGAAC while its reverse primer was GAAGGAAGCCTGGGTGAA; the forward primer for HAX1 was GGCTTGCTTTCCGGTAG while its reverse primer was ACGCGAACCTTTGAACC; the forward primer for GNPDA1 was GCAACAGACACTGCCACA while its reverse primer was CAGGAGAGCGGGACACT; and, the forward primer for HDLBP was ACAGGGAAAGAAAGCAAGG while its reverse primer was CAGATGGGGAAGAGGTGA. All experiments were done in duplicates. The Human Protein Atlas (HPA) database (https://www.proteinatlas.org/) was used to evaluate the protein expression levels of the five glycolysis-related genes in LIHC tissues, COAD tissues and corresponding normal tissues (Additional file 1: Appendix S1).

### Statistical analysis

We used the median value of the risk score to assign the 296 patients into high- and low-risk groups. Kaplan–Meier curves and log-rank methods were used to assess the prognostic significance of the risk score. Next, differential expression of the selected genes was examined and classified into high- and low-risk groups according to the median risk score. The receiver operating characteristic (ROC) curve analysis was performed to assess the sensitivity and specificity of prognostic prediction while the univariate and multivariate Cox analyses were performed to determine whether the risk score was an independent indicator of other clinical characteristics, including age, gender, grade and stage. Hazard ratios (HRs) and 95% confidence intervals (CIs) were used to assess the relative risk. Moreover, survival curves of clinic-pathologic characteristics and model validation between the two groups were created using the Kaplan-Meier method. *p* ≤ 0.05 was considered statistically significant. All statistical analyses were performed using the R 3.6.3 and GraphPad Prism 7 softwares.

## Results

### Differently expressed glycolysis-related genes in Asian gastrointestinal cancer patients

**Fig. 1:**
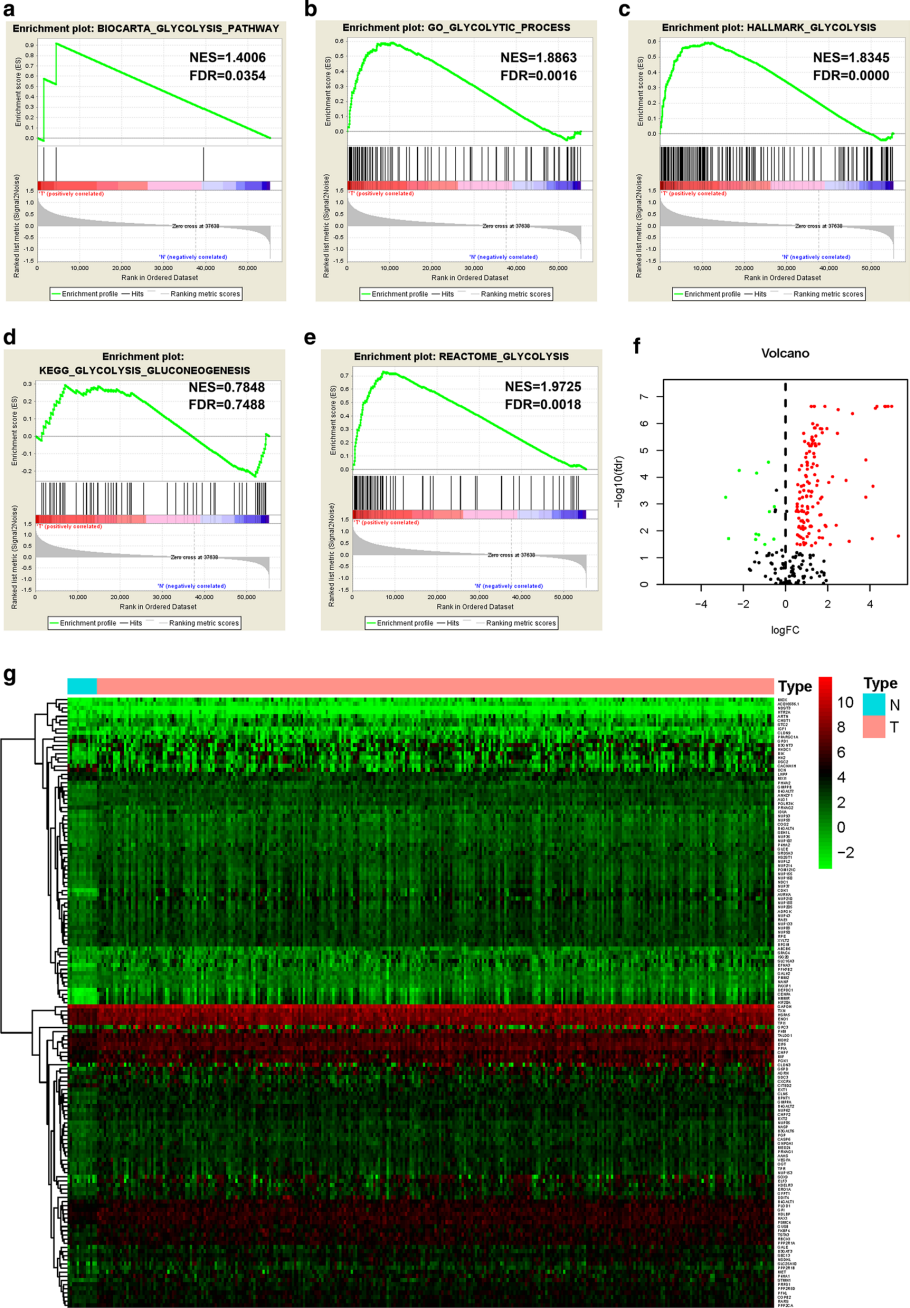
Performance of GSEA and differential expression analysis of glycolysis-related genes based on the Asian GI cancer patients of TCGA datasets. Enrichment analysis was performed on the selected gene sets, including BIOCARTA_GLYCOLYSIS_PATHWAY (**a**), GO_GLYCOLYTIC_PROCESS (**b**), HALLMARK_GLYCOLYSIS (**c**), KEGG_GLYCOLYSIS_GLUCONEOGENESIS (**d**) and REACTOME_GLYCOLYSIS (**e**). Volcano map (**f**) and heatmap (**g**) of glycolytic genes expressed differentially in tumor and normal tissues

We obtained the mRNA expression profiles and clinical data for 296 Asian GI cancer patients from the TCGA database. Compared to normal tissues, GSEA revealed that glycolysis-related gene sets were significantly enriched in Asian GI tumor tissues (Fig. 1a-e). Using |log2 (Fold Change)| > 0 and *p* < 0.05, we finally identified 19 up-regulated and 138 down-regulated glycolysis-related genes in Asian GI tumor and non-tumor tissues (Fig. 1f). Heat maps were established to show the differentially expressed genes between the tumor and normal groups (Fig. 1g).

### Construction of a risk score formula as an indicator of prognosis with the univariate Cox regression analysis

**Fig. 2:**
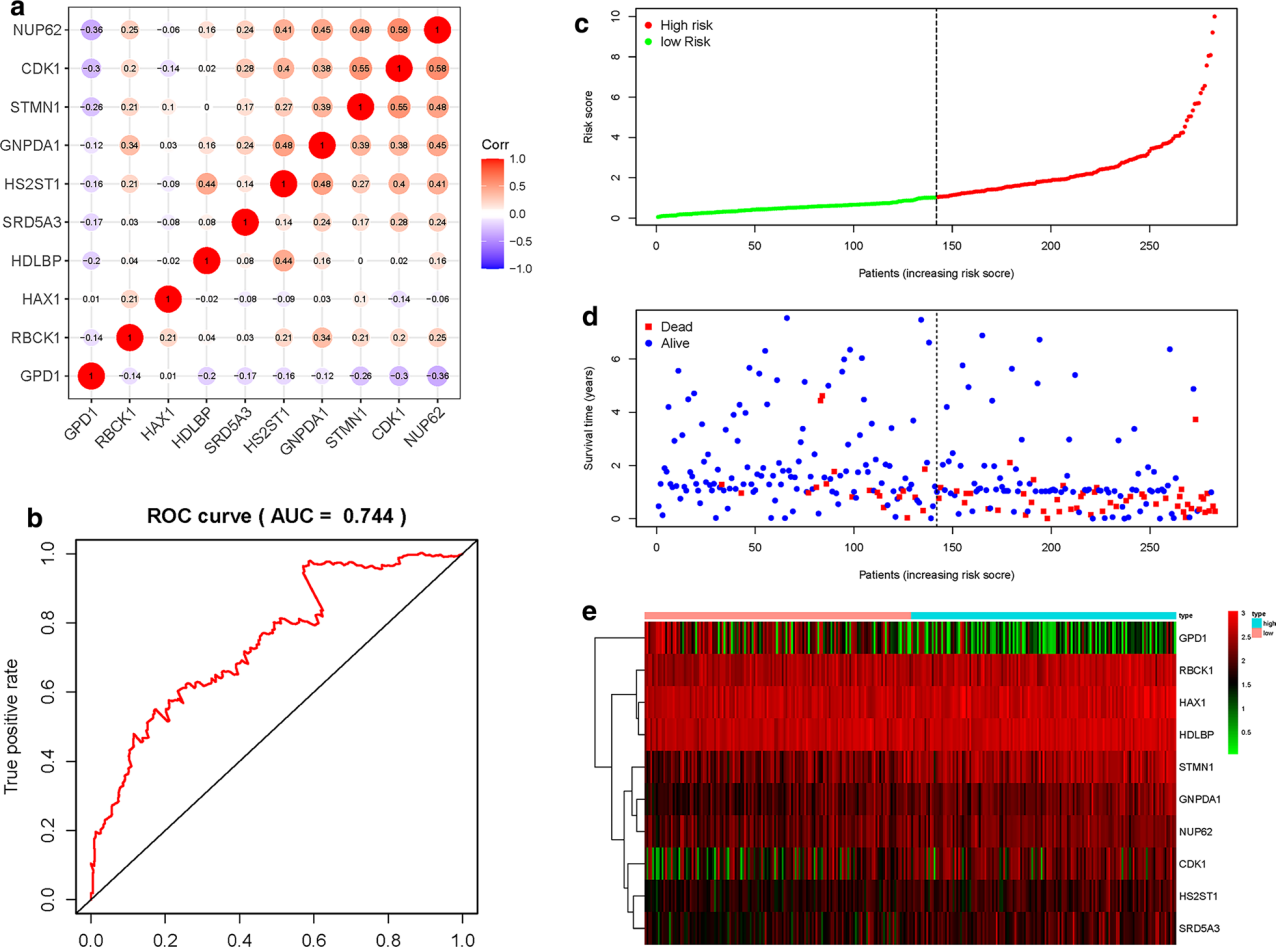
Construction of a risk score formula as an indicator of prognosis with the univariate Cox regression analysis in Asian GI cancer patients. **a** Correlations of significant differentially expressed genes. **b** Receiver operating characteristic (ROC) curve analysis of glycolysis-related model at 5 years. **c** Risk score distribution in each Asian gastrointestinal (GI) cancer patient. **d** Survival in days of GI cancer patients in ascending order of risk scores. (F) Heatmap of the expression profile of the 10 glycolysis-related genes

We used the univariate Cox regression analysis to screen and identify the genes associated with prognosis and survival. Ten mRNAs (RBCK1, HS2ST1, GPD1, SRD5A3, HAX1, GNPDA1, CDK1, NUP62, HDLBP and STMN1) were screened and identified as independent potential factors associated with poor prognosis. The candidate mRNAs were classified into two types: a risk type (RBCK1, HS2ST1, SRD5A3, HAX1, GNPDA1, HDLBP and STMN1) with HR > 1, which was associated with poor prognosis and a protective type (GPD1, CDK1, NUP62,) with HR < 1, which was associated with good prognosis (data not shown). Pearson correlation coefficients for the 10 mRNAs revealed strong correlations between: CDK1 and NUP62; STMN1 and NUP62; GNPDA1 and NUP62; HS2ST1 and NUP62; GPD1 and NUP62; STMN1 and CDK1; GNPDA1 and CDK1; HS2ST1 and CDK1; GNPDA1 and STMN1; HS2ST1 and GNPDA1; RBCK1 and GNPDA1 as well as between HDLBP and HS2ST1, with a correlation coefficient greater than 0.3 (Fig. 2a). Using the expression levels of the 10 mRNAs together with their regression coefficients as assessed by multivariate Cox analysis, a prognostic risk score formula was established: Risk score = 0.3459 × expression of RBCK1 + 0.5377 × expression of HS2ST1 – 0.3413 × expression of GPD1 + 0.3543 × expression of SRD5A3 + 0.6679 × expression of HAX1 + 0.4753 × expression of GNPDA1 – 0.3001 × expression of CDK1 – 0.6322 × expression of NUP62 + 0.5384 × expression of HDLBP + 0.3651 × expression of STMN1. ROC curve analysis of the mRNA signature was 0.744 at 5 years, indicating a good performance in predicting the prognosis of GI cancers (Fig. 2b). Subsequently, patients were assigned into low- and high-risk groups based on the median value of risk scores (Fig. 2c). We evaluated the survival times of patients in the high- and low- risk groups and found that mortality rates for patients with high-risk scores were higher than those with low-risk scores (Fig. 2d). Heatmap analysis was performed to reveal the expression profiles of the 10 mRNAs. Based on the survival risk score of the 10-mRNA expression, patients were divided into a low- or high-risk groups using the median risk score (Fig. 2e).

### Construction of the five-gene signature as an indicator for prognosis

**Fig. 3:**
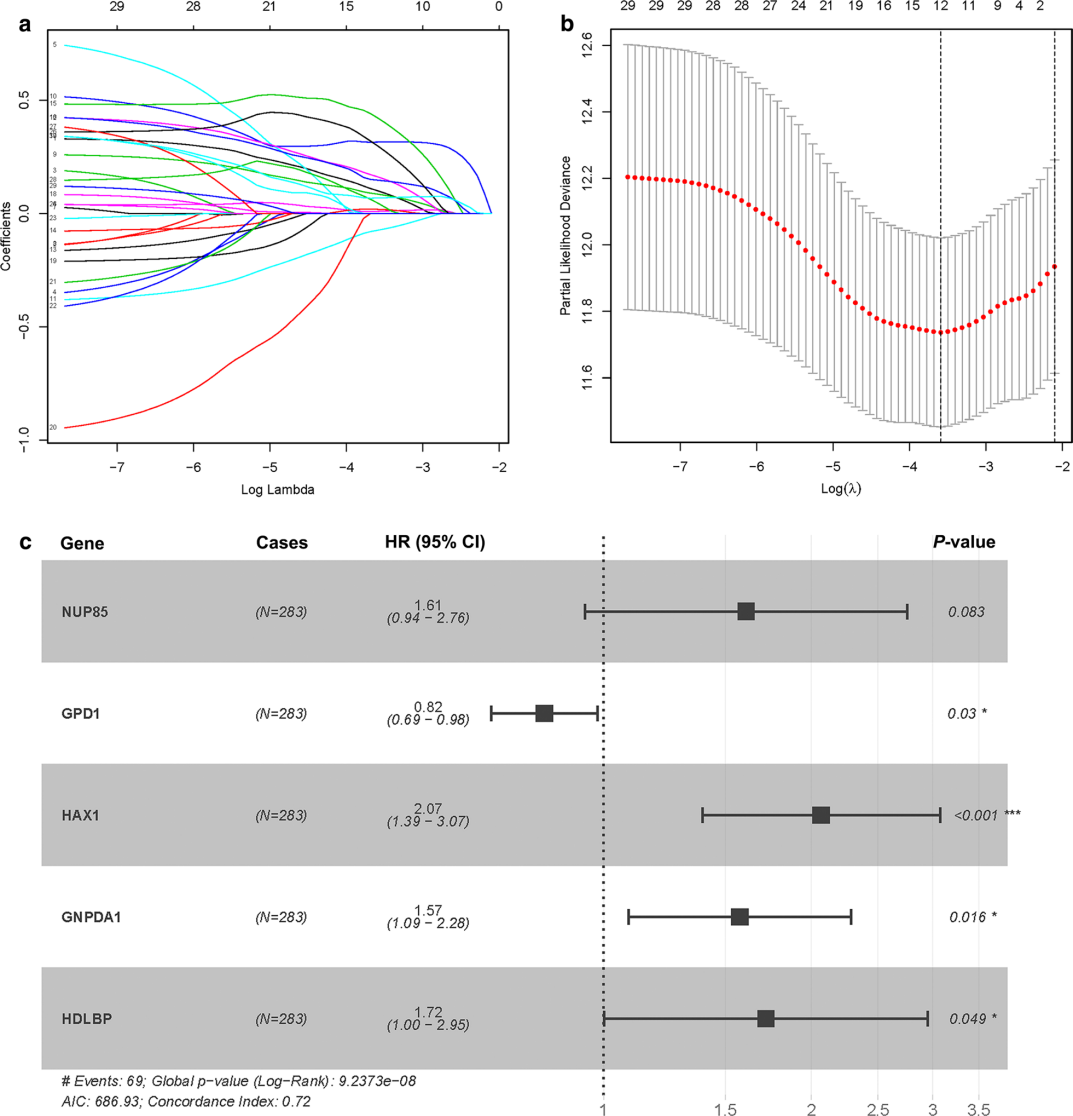
LASSO Cox regression model construction. **a** Curves represent regularization paths of LASSO coefficients. **b** Partial likelihood deviance as a function of regularization parameter λ in the TCGA dataset. **c** Forest plot describing the relationship between the five glycolysis-related gene expression and prognosis in GI cancer, *p < 0.05 and ***p < 0.001

**Fig. 4:**
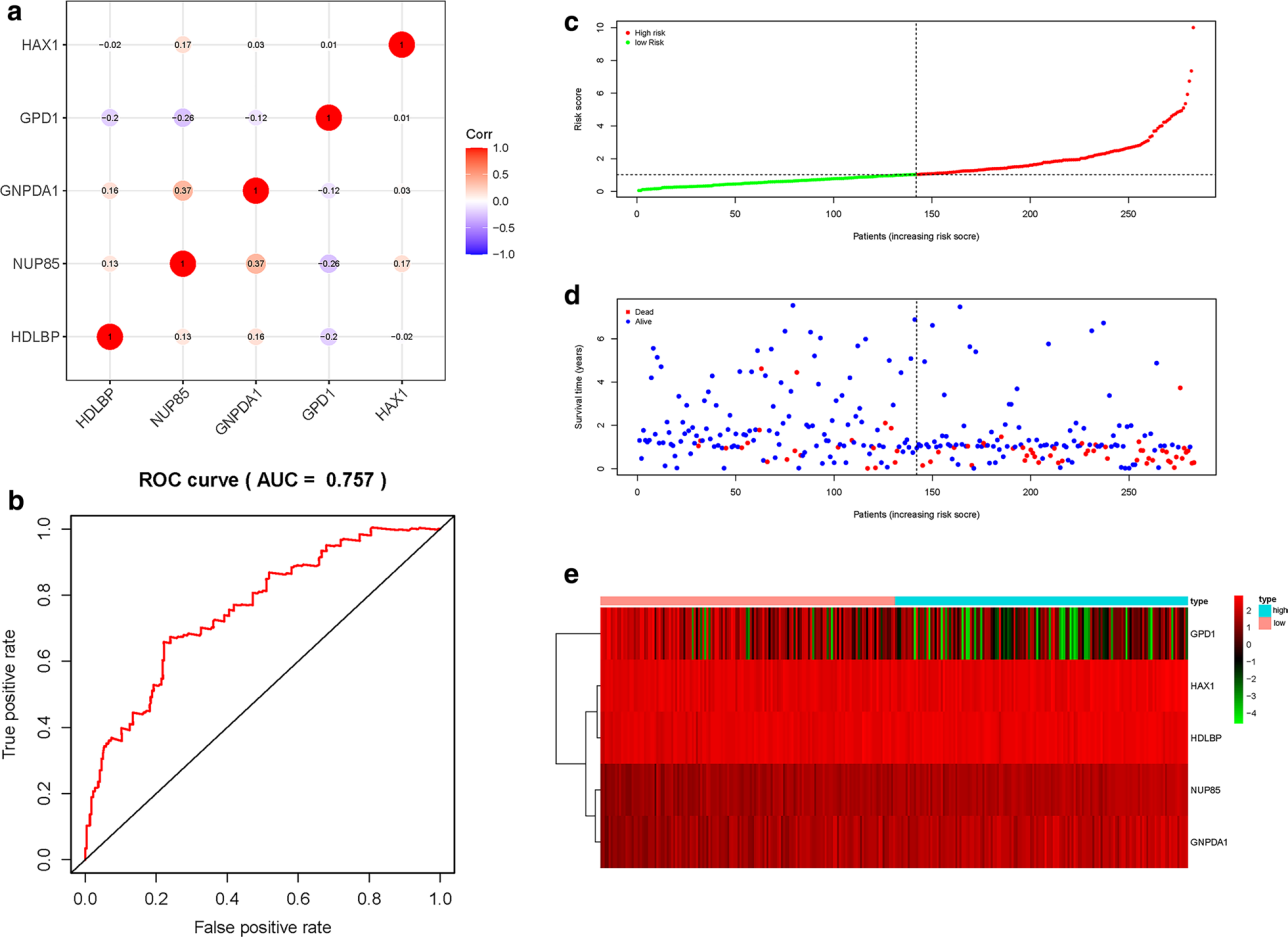
Construction of a risk score formula as an indicator of prognosis with the multivariate Cox regression analysis. **a** Correlations of significant differentially expressed glycolysis-related genes. **b** ROC curve analysis of glycolysis-related model at 5 years. **c** Risk score distribution in each Asian GI cancer patient. **d** Survival in days of GI cancer patients in ascending order of risk scores. **e** Heatmap of the expression profile of the five glycolysis-related genes

LASSO COX regression analysis was performed to optimize the prognostic model and prevent overfitting (Fig. 3a). It was found that the regression coefficient for each gene and the model achieved the best performance (Fig. 3b). Finally, five genes were screened as independent potential prognostic factors for OS. NUP85, HAX1, GNPDA1 and HDLBP with HR > 1 were considered as risk genes, whereas GPD1 with HR < 1 was considered as a protective gene (Fig. 3c and Table 2). Similarly, we calculated the Pearson correlation coefficients for the five mRNAs and found strong correlations between GNPDA1 and NUP85, with a correlation coefficient greater than 0.3 (Fig. 4a). Moreover, we established a prognostic risk score formula as previously described: Risk score = 0.4761 × expression of NUP85 – 0.1974 × expression of GPD1 + 0.7262 × expression of HAX1 + 0.4541 × expression of GNPDA1 + 0.5417 × expression of HDLBP. The ROC curve analysis with a five-mRNA signature was 0.757 at 5 years, better than 0.744 of the previous model, indicating that this model has a high sensitivity and specificity in predicting survival outcomes in Asian GI cancer patients (Fig. 4b). Based on the median value of the risk score, patients were assigned into low- and high-risk groups (Fig. 4c). Analysis of survival outcomes of patients in the high- and low- risk groups showed that mortality rates for patients in the high-risk group were higher than those in the low-risk group (Fig. 4d). Then, heatmap analysis was performed to reveal the expression profiles of the five genes in the low- or high-risk group (Fig. 4e).

**Table 2:** The information of five prognostic mRNAs weighted by its multivariable LASSO regression coefficient, which importantly associated with overall survival in Asian patients with gastrointestinal cancer

mRNA	Ensemble ID	Location	Risk coefficient	HR (95% CI)	*P* value
NUP85	ENSG00000125450	Chromosome 17: 75,205,557-75,235,758	0.4761	1.6097 (0.9400-2.7565)	0.0828
GPD1	ENSG00000167588	Chromosome 12: 50,103,982-50,111,313	-0.1974	0.8208 (0.6871-0.9805)	0.0295
HAX1	ENSG00000143575	Chromosome 1: 154,272,589-154,275,875	0.7262	2.0672 (1.3904-3.0734)	0.0003
GNPDA1	ENSG00000113552	Chromosome 5: 141,991,749-142,013,041	0.4541	1.5748 (1.0864-2.2826)	0.0165
HDLBP	ENSG00000115677	Chromosome 2: 241,227,264-241,317,061	0.5417	1.7189 (1.0023-2.9479)	0.0490

### Relationships between risk scores and clinical characteristics

**Fig. 5:**
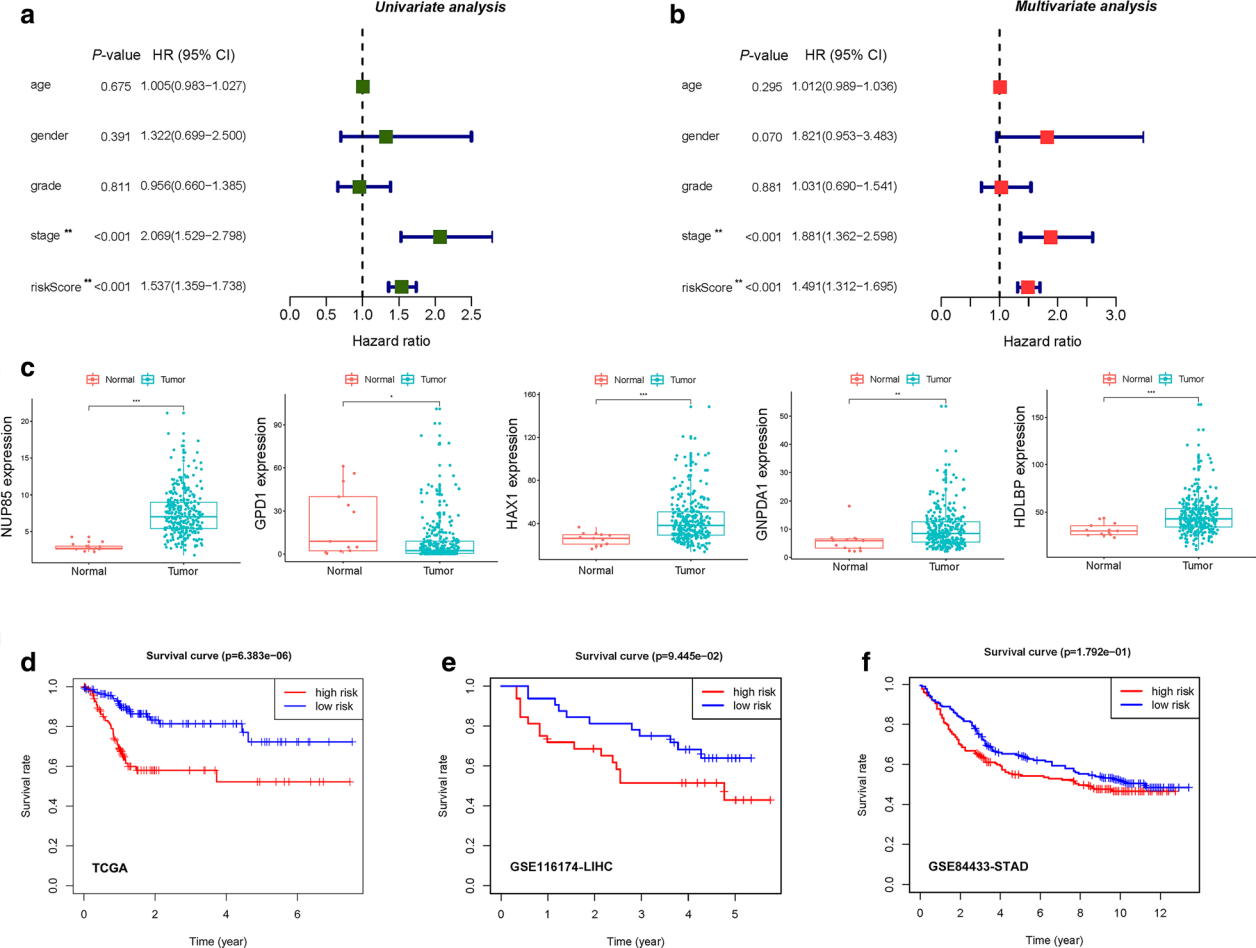
Analysis of risk factors and survival analysis plotted by Kaplan-Meier curves. **a** Univariate Cox regression analysis of the relationship between glycolysis risks core and clinical characteristics. **b** Multivariate Cox regression analysis of the relationship between glycolysis risks core and clinical characteristics. **c** Expression of the five mRNAs in GI tumor tissues and normal tissues. (*p < 0.05, **p < 0.01, ***p < 0.001). **d** Kaplan-Meier survival curves showing the overall survival probability stratified by the low- and the high-risk groups in the TCGA dataset. **e** Kaplan-Meier survival curves showing the overall survival probability stratified by the low- and the high-risk groups in the GSE116174 dataset. **f** Kaplan-Meier survival curves showing the overall survival probability stratified by the low- and the high-risk groups in the GSE84433 dataset

**Fig. 6:**
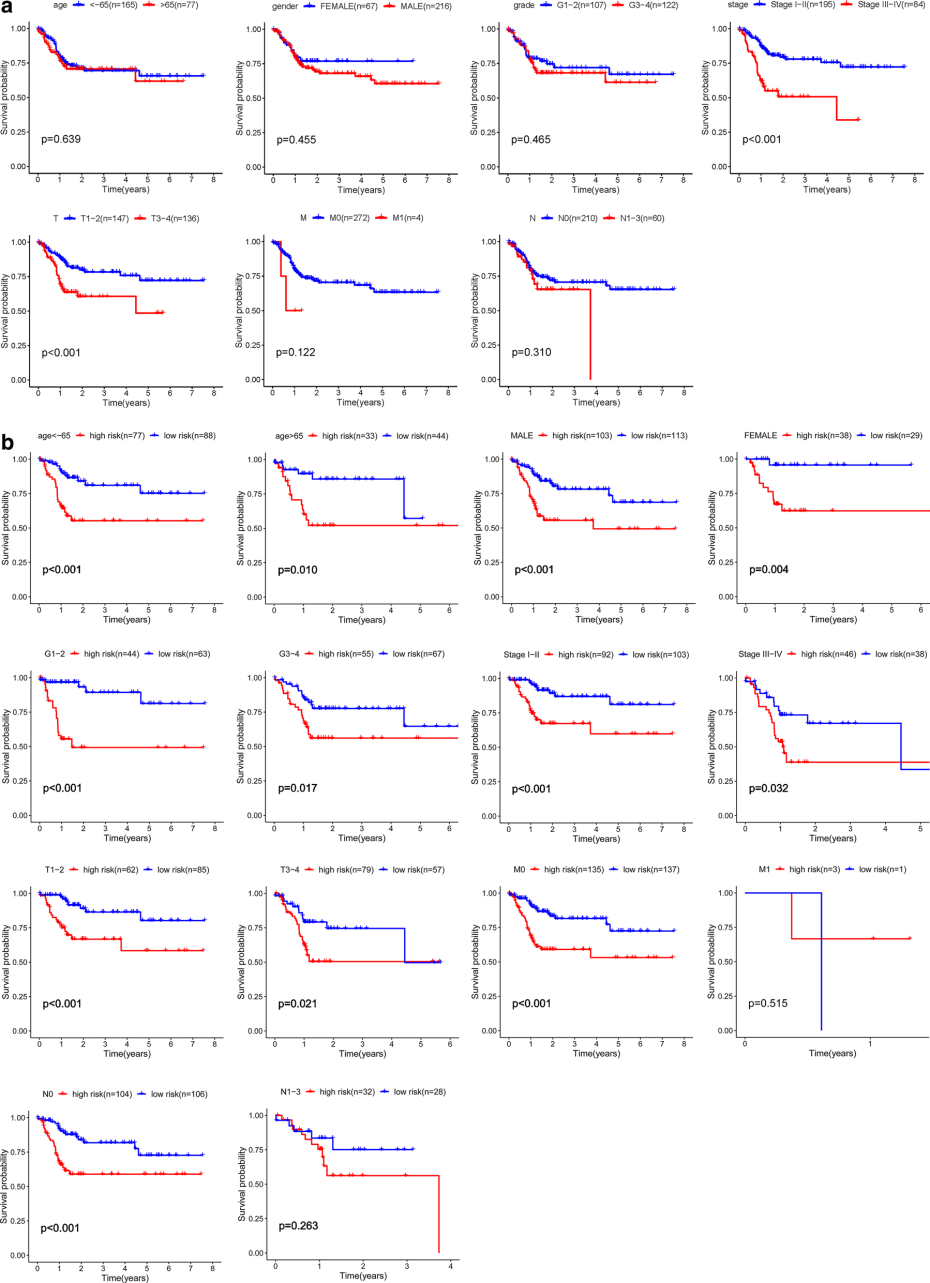
Kaplan-Meier survival analysis for Asian GI cancer patients in TCGA dataset. **a** Relationship between the clinical features and survival rate. **b** Prognosis of risk scores for the Asian GI cancer patients categorized by the clinical feature

We performed the univariate and multivariate Cox regression analyses to evaluate the effects of risk scores and other clinical parameters, including age, gender, grade and stage, on the prognostic value. The univariate Cox regression analysis showed that the five-gene risk score (HR = 1.537, 95% CI 1.359-1.738, *p* < 0.001) and stage (HR = 2.069, 95% CI 1.529-2.798, *p* < 0.001) correlated with the prognosis of GI cancer patients (Fig. 5a). In addition, the risk score and stage were found to be independent prognostic indicators (*p* < 0.001, Fig. 5b). These findings imply that the model can efficiently predict the prognosis of GI cancer with glycolysis-related gene risk score as an independent indicator (Fig. 5a, b). Furthermore, the expression levels of four mRNAs (NUP85, HAX1, GNPDA1 and HDLBP) were found to be elevated while that of GPD1 in tumors from the TCGA database was suppressed (Fig. 5c), consistent with our previous results. Then, we calculated the five-gene-based risk score for GI cancer patients. Patients in the high-risk group showed significantly poor OS than those in the low-risk group (*p* < 0.001) (Fig. 5d). To validate the generated prognostic model, GSE116174 and GSE84433 datasets were downloaded from Gene Expression Omnibus (GEO) and utilized as external samples. It was revealed that the survival and prognosis of Asian liver cancer patients and gastric cancer patients in the high-risk group were worse (Fig. 5e, f). Previous univariate and multivariate Cox regression analyses showed that tumor stage was correlated with the prognosis of GI cancer patients. Next, the Kaplan–Meier curve analysis was used to analyze colon cancer microarrays of Asian populations. We found that patients in stage III + IV (*p* < 0.001) and in T3-4 (*p* < 0.001) had poorer prognostic outcomes, consistent with our previous results (Fig. 6a). This model showed a good performance in stratifying age ≤ 65 (*p* < 0.001) and > 65 (*p* = 0.010), male (*p* < 0.001) and female (*p* = 0.004), grade G1-2 (*p* < 0.001) and G3-4 (*p* = 0.017), clinical-stage I-II (*p* < 0.001) and III-IV (*p* = 0.032), T1-2 (*p* < 0.001) and T3-4 (*p* = 0.021), M0 (*p* < 0.001) and N0 (*p* < 0.001) (Fig. 6). Analogous to the aforementioned results, the high-risk group in both subgroups was associated with worse OS, especially in patients without lymph node and/or distant metastasis. Overall, these results confirmed that the five-gene expression signature was an independent risk factor for predicting the survival of GI cancer patients in the Asian population.

### Expression levels of glycolysis-related genes in clinical tissue samples

**Fig. 7:**
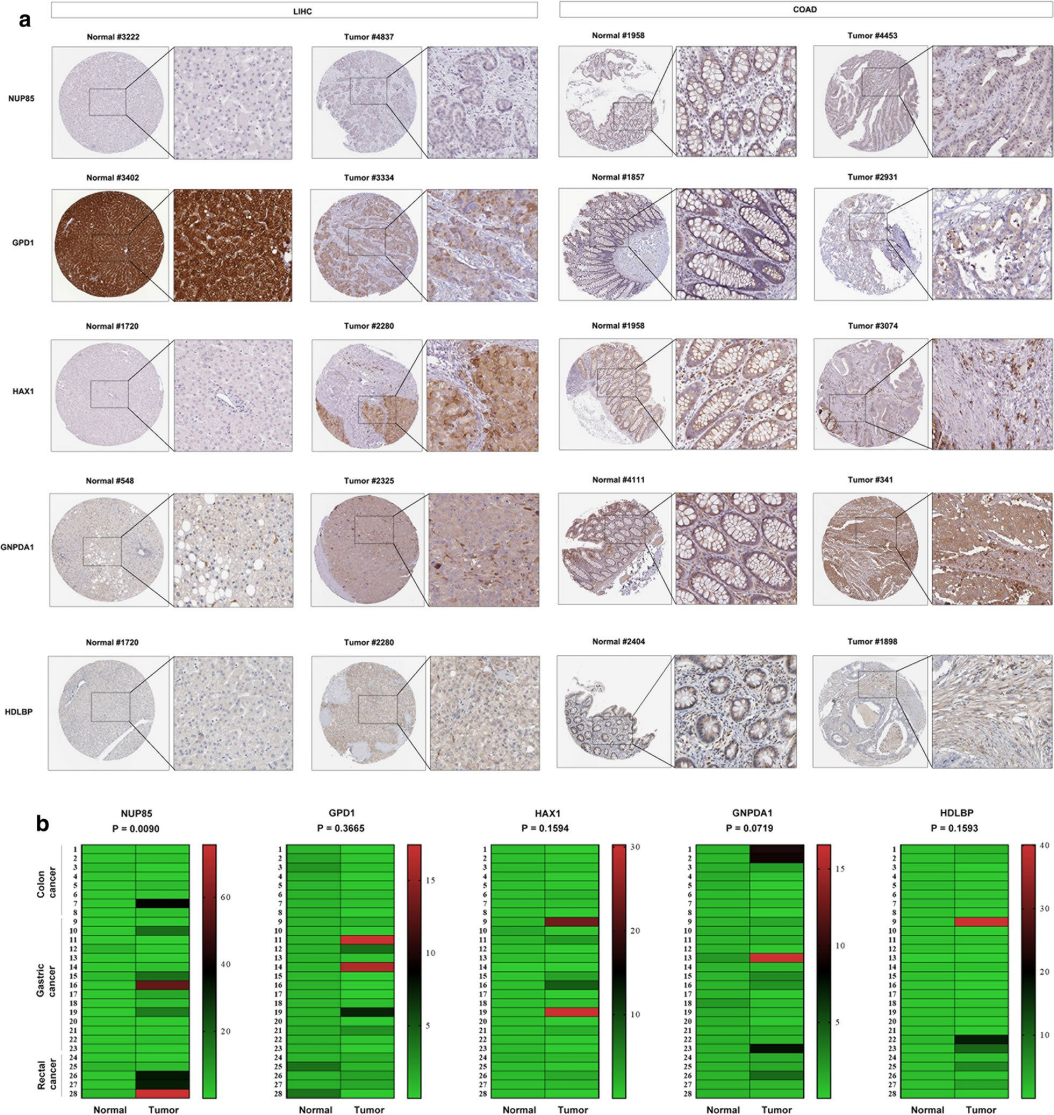
Expression levels of genes in clinical tissue samples. **a** Representative images of immunohistochemistry staining of the five glycolysis-related genes from the Human Protein Atlas (HPA) database, including LIHC and COAD. **b** Expression of the five glycolysis-related genes in 28 paired clinical samples, including 8 paired COAD tissues, 5 paired READ tissues and 15 paired STAD tissues, using qRT-PCR to examine

The HPA database was used to evaluate protein expression levels for NUP85, GPD1, HAX1, GNPDA1 and HDLBP in LIHC and COAD tissues compared to their expression in normal tissues. The NUP85, HAX1, GNPDA1 and HDLBP protein levels were significantly elevated in tumor tissues compared to normal samples, while GPD1 was significantly down-regulated in tumor tissues (Fig. 7a). In addition, NUP85, HAX1, GNPDA1 and HDLBP exhibited elevated mRNA expression levels in GI tumor tissues when compared to the adjacent non-tumor tissues, whereas GPD1 expression was suppressed in GI tumor tissues compared to the non-tumor tissues (Fig. 7b).

## Discussion

A GI tumor is a type of tumor that occurs in complex digestive organs and whose biodiversity as well as tumor characteristics are inconsistent [37]. It mainly includes liver hepatocellular carcinoma (HCC), stomach adenocarcinoma, esophageal carcinoma, pancreatic adenocarcinoma, colon adenocarcinoma, cholangiocarcinoma and rectal adenocarcinoma. The prevalence of HCC is higher in Asia than in America and Europe. About 78% of the global HCC cases are reported in Asian countries, with China accounting for about 55% of the global HCC cases [38]. GI cancers are the most common malignancies in Asia, especially in China and Japan [1]. Due to its importance and superior therapeutic efficacies, gene therapy is widely being evaluated [39]. Identifying effective biomarkers to construct a prognostic model is of great clinical significance in informing the clinical decision-making process. Several predictive models for patient survival rates have been identified, however, they all have limitations. For example, in the autophagy-related gene prognosis prediction model, autophagy is a double-edged sword in various tumors, promoting as well as inhibiting cancer progression [40]. Therefore, the expression levels of autophagy related genes are unreliable. Similarly, in the immune response-related gene prognostic model, the established tumors often induce immune tolerance at an early stage of tumorigenesis, resulting in abnormal immune responses [41]. Glycolysis is the main energy source for cancer cells and the primary energy source for tumor invasion [42]. Studies have reported that glycolysis is a potential therapeutic and prognostic target for cancers [43–48]. Considering its role in cancer, constructing a glycolysis associated gene risk signature may be advantageous for the accurate diagnosis, therapy and prognosis of GI cancers. In addition, the prognostic significance of glycolysis-related genes in Asian GI cancers has not been reported.

**Fig. 8:**
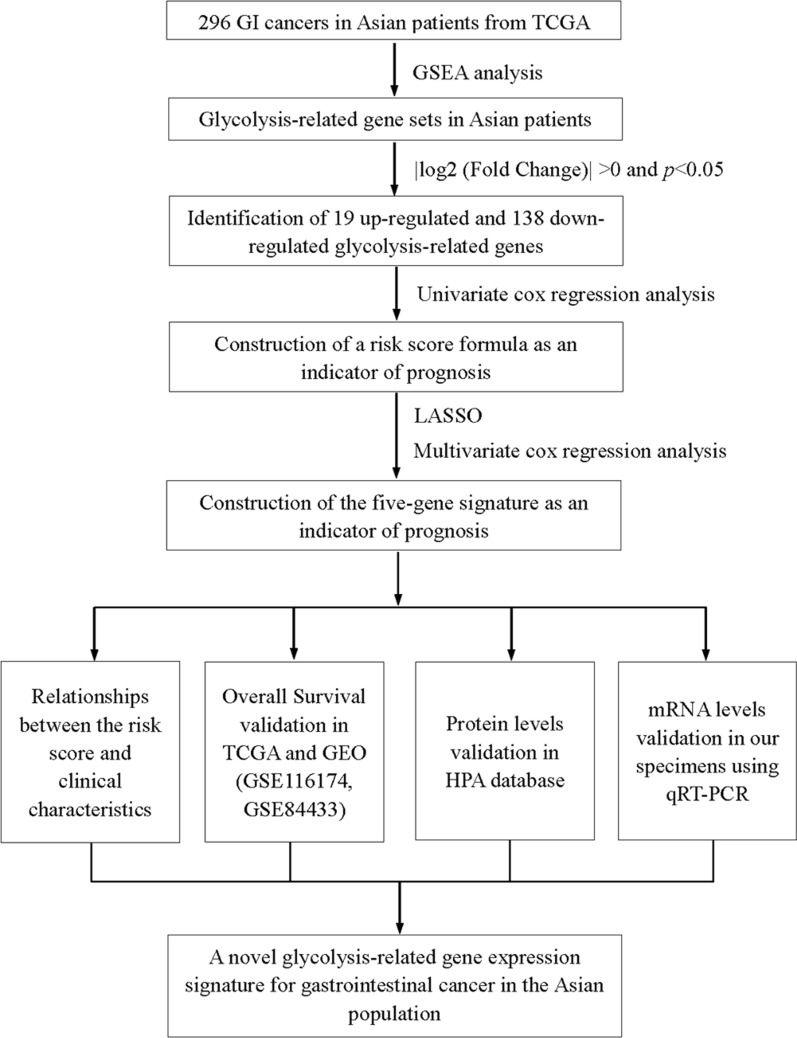
A flowchart of the data analysis procedures

We identified five novel glycolysis-associated genes (NUP85, GPD1, HAX1, GNPDA1, and HDLBP) in GI tumor and normal tissues. GPD1 was fond to be a positive prognostic gene, while NUP85, HAX1, GNPDA1, HDLBP were negative prognostic genes. The nuclear pore complex (NPC) is a combination of macromolecules that cross the nuclear membrane to form a selective barrier between the nucleus and the cytoplasm [49]. The central channel of NPCs is filled with nucleoporins (NUPs), which can build a size-selective diffusion barrier for macromolecules larger than 40 kDa, while providing binding sites for nuclear transport receptors (nuclear transporters, importins and exportins), thereby transporting signal-carrying cargo across the NPC. NUP85 is an important member of the NPC outer ring [50]. It is postulated that dysregulated NUP85 functions may lead to tissue homeostasis imbalance. We found elevated NUP85 expression levels in tumors from the TCGA database in the Asian population, suggesting its possible involvement in the development of Asian GI cancers. It has been reported that targeting NUP85 in pancreatic cancer cells inhibits their invasiveness and metastasis. Glycerol-3-phosphate dehydrogenase 1 (GPD1) is an NAD+/NADH dependent enzyme, which plays an important role in the cytoplasm as a glycerol phosphate shuttle [51]. Abnormal GDP1 expression may exert adverse effects on human health. GPD1 expression has been shown to be activated in early tumor development stages, such as those of glioblastoma [52]. However, GPD1 may exert an antitumor effect [53–55]. As a central component of lipid metabolism and synthesis, abnormal GDP1 activity can induce multiple digestive system diseases [56, 57]. Therefore, the role of GPD1 in GI tumors is worthy of attention. We found that GDP1 expression levels in GI tumors was relatively low, which may be related to GPD1 deficiency and its effect on gluconeogenesis. In tumor sites, hematopoietic substrate-1-associated protein X-1 (HAX-1) is highly expressed during neovascularization [58]. HAX-1 promotes the migration and invasion of carcinoma cells by disrupting apoptotic responses [58–60]. We also confirmed that elevated HAX-1 expression levels are closely correlated with tumor development. Glucosamine-6-phosphate isomerase 1 (GNPDA1) can catalyze the conversion of glucosamine 6-phosphate to fructose 6-phosphate and thereby increase the raw materials for glycolysis [61–63], which enhances cancer progression. GNPDA1 plays important roles in cell proliferation, migration and invasion [64, 65]. Elevated GNPDA1 expression levels are associated with poor prognosis in patients with HCC, pancreatic cancer and colorectal cancer [64, 66–68]. Furthermore, high-density lipoprotein binding protein (HDLBP), also known as vigilin, has been shown to play a significant role in cellular sterol metabolism in human atherogenesis [69]. Several studies have shown that vigilin is highly expressed in multiple cancers, including gastric cancer, suggesting it may be a promoter for carcinogenesis [70–72]. In conclusion, these five genes are involved in the progression of GI cancers and were used to establish a five-gene prognostic signature. GI cancer patients in the high-risk group exhibited significantly poor prognosis than those in the low-risk group. Due to the biological functions of the five genes in carcinogenesis and the significant correlation with the prognosis of GI cancer patients, the five-gene signature is a novel biomarker that can be used to inform clinical decisions (Fig. 8).

However, this study is associated with several limitations. Our datasets were mainly from the TCGA database in the Asian population. Although we validated the expression levels of the identified genes in the collected tissues, only a small number of Asian patient samples were represented. Further validation of the five-gene expression signature in a large sample population is necessary. Meanwhile, in Asian people of different ancestry, genetic variation should be considered as a correction factor. Besides, this study provides the possibility that the five-gene expression signature may function as a therapeutic and prognostic target, which was merely analyzed through available retrospective data. The underlying mechanisms of the five-gene expression signature in cancer progression should be elucidated through functional experiments. In recent years, studies have reported that non-coding RNA plays an important role in the initiation and progression of cancer. Aberrant expression of non-coding RNAs have been found to be involved in the regulation glycolysis associated genes [73–77]. Thus, further studies on the non-coding RNAs that are associated with glycolysis in GI cancer are also necessary. In summary, we identified and validated a glycolysis associated five-gene risk signature (NUP85, GPD1, HAX1, GNPDA1 and HDLBP) that can predict the OS of GI Asian cancer patients. This five-gene signature can be used as a novel tool in clinical practice. More studies should evaluate the roles of these genes in Asian GI cancers, which can provide the theoretical basis for clinical practice. Furthermore, more data is be needed to validate the general applicability of this signature in clinical decisions.

## Conclusions

We systematically established five glycolysis-related genes (NUP85, GPD1, HAX1, GNPDA1 and HDLBP) in Asian GI cancers. Moreover, we established a five-gene expression signature and showed that the predictive model can independently predict the OS of Asian GI cancer patients by combining molecular signatures and clinical characteristics.

## Supplementary Information

**Additional file 1: Appendix S1.** Basic information about the tumor samples we used in this study.

## Data Availability

The data sets used and/or analyzed during the current study are publicly available data from The Cancer Genome Atlas (TCGA), Gene Expression Omnibus (GEO), and Human Protein Atlas. The figures and materials supporting the conclusions of this article are included within the article.

## Abbreviations

GI cancer: Gastrointestinal cancer
TCGA: The Cancer Genome Atlas
LIHC: Liver hepatocellular carcinoma
STAD: Stomach adenocarcinoma
ESCA: Esophageal carcinoma
PAAD: Pancreatic adenocarcinoma
COAD: Colon adenocarcinoma
CHOL: Cholangiocarcinoma
READ: Rectum adenocarcinoma
GSEA: Gene Set Enrichment Analysis
ROC: Receiver operating characteristic
NUP85: Nucleoporin 8
HAX1: HCLS1 associated protein X-1
GNPDA1: Glucosamine-6-phosphate deaminase 1
HDLBP: High density lipoprotein binding protein
GPD1: Glycerol-3-phosphate dehydrogenase 1
LASSO: Logistic least absolute shrinkage and selection operator
OS: Overall survival
MSigDB: Molecular signature database
FDR: False discovery rate
HR: Hazard ratio
qRT-PCR: Quantitative real-time PCR
RNA-seq: RNA sequencing
